# Mental Health and Kidneys: The Interplay Between Cognitive Decline, Depression, and Kidney Dysfunction in Hospitalized Older Adults

**DOI:** 10.3390/jcm14124120

**Published:** 2025-06-10

**Authors:** Diana Moldovan, Ina Kacso, Lucreția Avram, Dana Crisan, Ariana Condor, Cosmina Bondor, Crina Rusu, Alina Potra, Dacian Tirinescu, Maria Ticala, Yuriy Maslyennikov, Andrada Bărar, Alexandra Urs, Valer Donca

**Affiliations:** 1 Department of Nephrology, Faculty of Medicine, “Iuliu Hatieganu” University of Medicine and Pharmacy Cluj-Napoca, 400012 Cluj-Napoca, Romania; maria.kacso@umfcluj.ro (I.K.); claudia.rusu@umfcluj.ro (C.R.); alina.potra@umfcluj.ro (A.P.); tirinescu.dacian@umfcluj.ro (D.T.); cosa.maria@umfcluj.ro (M.T.); maslyennikov_yuriy@elearn.umfcluj.ro (Y.M.); barar_andrada_alina@elearn.umfcluj.ro (A.B.); alexandra.urs@elearn.umfcluj.ro (A.U.); 2 Emergency County Hospital, 400006 Cluj-Napoca, Romania; 3 Department of Geriatrics—Gerontology, Faculty of Medicine, “Iuliu Hatieganu” University of Medicine and Pharmacy Cluj-Napoca, 400012 Cluj-Napoca, Romania; valer.donca@umfcluj.ro; 4 Clinical Municipal Hospital, 400139 Cluj-Napoca, Romania; crisan.dana@umfcluj.ro (D.C.); ariana_condor@yahoo.com (A.C.); 5 Department of Internal Medicine, 5th Medical Clinic, Faculty of Medicine, “Iuliu Hatieganu” University of Medicine and Pharmacy Cluj-Napoca, 400012 Cluj-Napoca, Romania; 6 Department of Medical Informatics and Biostatistics, “Iuliu Hatieganu” University of Medicine and Pharmacy Cluj-Napoca, 400349 Cluj-Napoca, Romania; cbondor@umfcluj.ro

**Keywords:** chronic kidney disease, albuminuria, cognitive impairment, depression, older population

## Abstract

**Background:** As societies rapidly age, the prevalence of mental health disorders and chronic kidney disease (CKD) is simultaneously rising, and data on the link between these conditions remain inconclusive. This study aimed to investigate the associations among cognitive impairment, depression, and kidney involvement in elderly patients. **Methods:** A cross-sectional analysis was conducted among hospitalized patients aged ≥65 years. Standardized tools such as the geriatric depression scale (GDS) and Montreal Cognitive Assessment (MoCA) were used to assess depression and cognitive impairment, and kidney function was evaluated using eGFR and albuminuria. Bivariate and multivariate logistic regressions were performed to identify associations. **Results:** The study population consisted of 719 participants with a median age of 80 years. Kidney and mental health issues were highly prevalent: CKD was identified in 59.4%, cognitive impairment in 74%, and depression in 61.9% of patients. Patients with CKD were older and exhibited lower MoCA scores (*p* = 0.001), higher GDS scores (*p* = 0.007), reduced albumin (*p* < 0.001), lower hemoglobin levels (*p* < 0.001), and elevated C-reactive protein (*p* < 0.001). Increased albuminuria was associated with poorer cognition (*p* < 0.001) but showed no correlation with GDS scores. Additionally, worse cognitive scores (*p* = 0.001) and increased depression symptoms (*p* < 0.001) were correlated with declining estimated glomerular filtration rate (eGFR). **Conclusions:** Cognitive impairment and depressive symptoms are highly prevalent among elderly hospitalized patients. Cognitive decline correlates with increased albuminuria and reduced eGFR, while depression worsens with declining kidney function. These findings highlight the complex interplay between renal health and neuropsychiatric conditions in aging populations.

## 1. Introduction

The prevalence of chronic kidney disease (CKD) is roughly 10% in the general population, with a substantial increase observed in older adults, affecting more than one-third of individuals over the age of 65. The augmented incidence of CKD in the elderly is attributable to the combination of age-related physiological changes, including a decline in renal function and regenerative capacity, along with the accumulation of multiple comorbidities [1]. Albuminuria is an early indicator of CKD, but also an important independent predictor of endothelial dysfunction as well as cardiovascular and all-cause mortality [2].

Cognitive dysfunction and depression are notable complications in CKD, and old age worsens the severity of these neurologic adverse outcomes [3].

Several factors contribute to cognitive decline in CKD patients, encompassing aging, anemia, oxidative stress, inflammation, malnutrition, and uremic toxins, as well as comorbidities like diabetes, hypertension, and cardiovascular disease [4]. CKD-related cognitive impairment can range from mild cognitive deficits to severe dementia, and it can substantially reduce quality of life and the ability of patients to manage their own care. Early detection and management of cognitive dysfunction in CKD are crucial to optimize patient outcomes and improve overall well-being [5]. Cognitive decline can be detected using specific tools; among them, the Montreal Cognitive Assessment (MoCA) has shown reliability, especially in older patients [6]. It can help identify people with mild and early signs of dementia, people at risk of Alzheimer’s disease, and screen for conditions like Parkinson’s disease, brain tumors, and substance abuse.

Depressive symptoms are prevalent in geriatric patients, especially those with chronic diseases. CKD, which frequently coexists in this demographic group, likely affects mental health status significantly. The severity of depression often increases as CKD progresses, having an estimated prevalence of approximately 20% in CKD patients. The interactions between depression and CKD are complex, bidirectional, and multifactorial, owing to the constant burden of dealing with a progressive disease as well as with symptoms such as dyspnea, pain, pruritus, fatigue, and sleep disorders [7,8], and with complications such as vascular and bone complications in advanced CKD [9]. Depressive moods were also associated with malnutrition in older adults, and the psychological burden is worsened by complex medical regimens and associated financial issues [10,11]. Social networks may shrink as physical limitations and demanding treatment regimens impose barriers to social interaction, perpetuating a cycle of isolation and cognitive decline that can amplify feelings of despair. Concerns about disease progression and the potential need for dialysis or transplantation can contribute to anxiety [12]. Frequent medical appointments may also exacerbate anxiety by serving as reminders of patient vulnerability and dependence on healthcare systems. Regular mental health screening is crucial to identify and manage depression and anxiety in patients with CKD, and the geriatric depression scale (GDS) displays good predictive value in the elderly [13]. Depression in CKD is associated with multiple unfavorable outcomes, including increased mortality and hospitalization rates, as well as poorer treatment compliance and quality of life. In a recent retrospective cohort study on over 25,000 adult individuals, the participants with both depression and proteinuria had a significantly higher risk of all-cause and cardiovascular mortality [14]. Pharmacological interventions for mental health conditions should be considered in light of altered pharmacokinetics and pharmacodynamics in patients with kidney dysfunction [15].

Understanding the relationship between albuminuria, eGFR, and neuropsychiatric involvement like cognitive impairment and depression can help improve outcomes in elderly patients. However, data on the link between kidney dysfunction and neuropsychiatric conditions in older adults remain limited and inconclusive.

The main goal of this study was to investigate the interrelationship between cognitive decline, depression, and kidney dysfunction among hospitalized older adults, in light of their shared pathophysiological and clinical pathways. Secondary objectives were to obtain data on the prevalence of cognitive decline and depression and to identify additional factors associated with mental health in our geriatric CKD population.

## 2. Materials and Methods

This study was conducted in the geriatric ward of a tertiary hospital. Eligible participants were aged 65 years or older and hospitalized for healthcare assessment. It was carried out on hospitalized patients older than 65 years who agreed to participate in all evaluations and follow the study protocol. A diagnosis of chronic kidney disease (CKD) was not required for inclusion; however, kidney function was assessed in all participants. We excluded the patients on dialysis, in intensive care, and with acute infections or terminal illnesses. Of the 806 patients invited to participate, 736 agreed and completed the assessments. A total of 17 patients were excluded due to incomplete data, resulting in a final sample of 719 patients. There was no loss to follow-up, as the study was cross-sectional and data were collected during hospitalization. We evaluated data related to inclusion and exclusion criteria, and we recorded demographic and clinical information such as age, gender, or presence of diabetes.

The kidney involvement was evaluated by albuminuria and kidney function. Urinary albumin over creatinine ratio (UACR) values allowed the inclusion of patients in one of the categories of albuminuria: A1, which is normal or mildly elevated (UACR < 30 mg/g); A2, moderately elevated (UACR 30–300 mg/g); and A3, severely elevated (UACR > 300 mg/g).

Kidney dysfunction was identified by measuring serum urea, creatinine, and uric acid. The estimated glomerular filtration rate (eGFR) was obtained from the CKD Epidemiology Collaboration (EPI) 2021 equation.

We also measured the hemoglobin (Hb), serum albumin, C-reactive protein (CRP), vitamin B12, and 25-OH vitamin D levels.

To measure cognitive function, we used the Montreal Cognitive Assessment (MoCA). The MoCA evaluates 7 domains of cognition (executive/visuospatial function, naming, attention, language, abstraction, recall, and orientation). A score of less than 26 indicates cognitive impairment. We included our patients in 2 categories: MoCA ≥ 26, meaning normal cognition, and MoCA < 26, meaning cognitive impairment. The MoCA has demonstrated high sensitivity (90%) and specificity (87%) for detecting mild cognitive impairment, with good internal consistency (Cronbach’s alpha = 0.83) in older populations [6].

The presence and severity of depression was assessed with the geriatric depression scale (GDS). The GDS is a screening tool used to identify depression in older adults. The GDS has shown good validity and reliability in elderly populations, with a reported sensitivity of 92% and specificity of 89% for major depression. Scores of 0 to 4 were considered normal, depending on age, education, and complaints; scores over 5 include mild, moderate, and severe depression. Our patients with GDS ≤ 4 were considered normal, and those with GDS > 4 had depression of varying degrees.

The relationships between cognitive impairment, depression, kidney function, and albuminuria were tested.

### Statistics

The characteristics of the study sample were reported using descriptive statistics (mean and standard deviation for continuous variables, proportion for categorical variables) stratified according to eGFR and UACR. For comparing the range and distribution for groups of numerical data, we used box-and-whisker plots. Stacked bar graphs were used to portray comparisons of total values across several categories.

Qualitative ordinal variables were described using the median and percentiles. Qualitative variables or ordinal variables with less than 7 categories were described with absolute and relative frequencies. The chi square or Fisher exact tests were used to evaluate the relation between qualitative variables.

Normally distributed data were presented as the mean and standard deviation (SD), and skewed variables as the median and interquartile range. Normality was assessed using Kolmogorov–Smirnov and Shapiro–Wilk tests. For continuous factors, the statistical comparison was performed using the *t* test or Mann–Whitney Rank Sum Test. Variables were compared by subgroups using Student’s *t*-tests in the case of equal and negative variances and using the Mann–Whitney test.

The relationship between GDS, MoCA, and renal parameters was explored using linear regression models. The models were then adjusted for potential confounders, selected on the basis of the clinical significance, prior knowledge, and results of the bivariate analysis. Because the variables of interest had multiple outliers, we used Spearman’s rank correlation to assess the strength and direction of the relationship between two quantitative, ranked variables.

A significance level of *p* < 0.05 was considered for all statistical tests. Variables with *p* < 0.05 in the bivariate analysis were subsequently included in the multivariate logistic regression model. The analysis was performed using Microsoft Excel and SPSS 25.0.

## 3. Results

The study population consisted of 719 patients. The median age was 80 years; 25% were in the interval 65–74 years, 50% belonged to the interval 75–85 years, and 25% were over 85 years old; 29.8% were males. CKD was present in 59.38% and diabetes in 34.3%. Mental health issues were highly prevalent. The number of patients with a MoCA score below 26 points, defined as cognitive impairment, was 532, corresponding to 74%. The number of patients with GDS scores over 4 points, defined as depression, was 445, corresponding to 61.9%.

Two groups were created and analyzed based on the presence of CKD. The first group included patients with CKD defined as eGFR < 60 mL/min/m^2^ or UACR ≥ 30 mg/g for more than 3 months. The second group included patients with eGFR ≥ 60 mL/min/m^2^ and UACR < 30 mg/g. When comparing the groups according to CKD presence, patients with CKD were older, with more females, and had lower MoCA (*p* = 0.001), lower albumin (*p* < 0.001), lower hemoglobin (*p* < 0.001)**,** higher GDS (*p* = 0.007), and higher CRP (*p* < 0.001). Table 1 illustrates the descriptive statistics and the comparison between the groups of patients with and without CKD.

**Figure 1:**
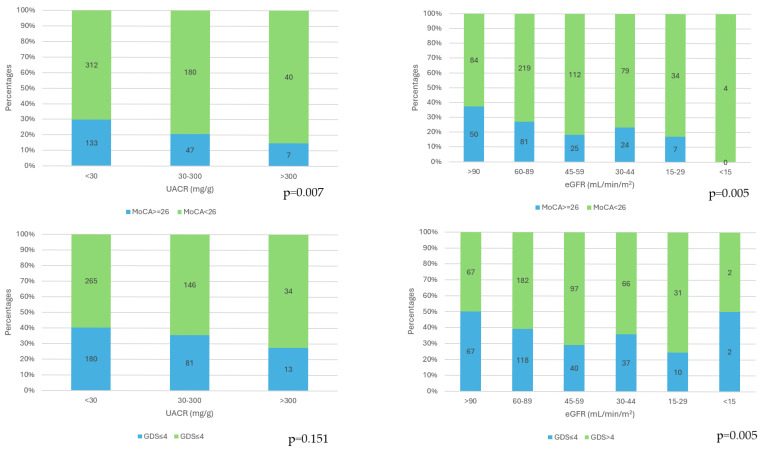
Distribution of cognitive impairment (MoCA scores < 26) in the three categories of albuminuria (*p* = 0.007) and in the five CKD stages (*p* = 0.005)**.** Distribution of depression (GDS scores > 4) in the three categories of albuminuria (*p* = 0.151) and in the five CKD stages (*p* = 0.005).

The cognitive impairment (MoCA scores < 26) frequences were significantly higher as the category of albuminuria increased (*p* = 0.007) and as the CKD stages increased (*p* = 0.005). The presence of depression (GDS scores > 4) was not influenced by the category of albuminuria (*p* = 0.151), and depression prevalence was significantly different in the CKD stages (*p* = 0.005), with the highest frequency in stage 4 (Figure 1).

**Figure 2:**
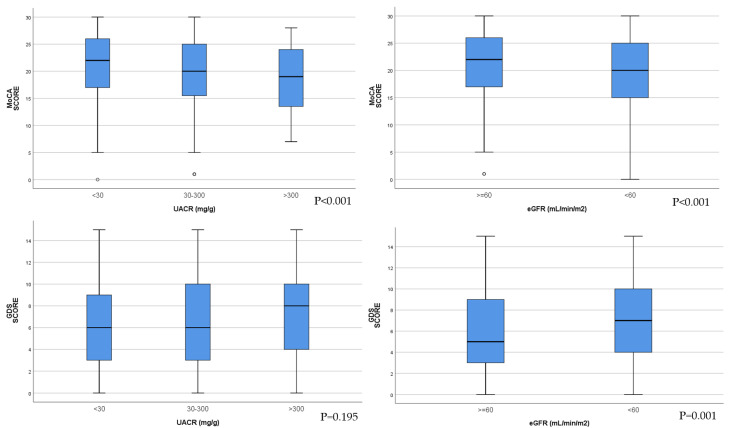
Comparison between MoCA scores in the three groups according to UACR (*p* < 0.001) and the two groups according to eGFR (*p* < 0.001). Comparison between GDS scores in the three groups according to UACR (*p* = 0.195) and the two groups according to eGFR (*p* = 0.001).

MoCA scores were significantly lower with the increase in albuminuria category. In the group of patients with eGFR < 60 mL/min/1.73 m^2^, MoCA scores were decreased compared with the group of patients with eGFR ≥ 60 mL/min/1.73 m^2^ (Figure 2).

There were no significant differences in GDS score between the three categories of patients according to albuminuria levels. GDS scores were significantly higher, indicating depression in the group of patients with eGFR < 60 mL/min/1.73 m^2^ (Figure 2).

The correlation studies identified factors associated with MoCA score and with GDS score. All correlations are illustrated in Table 2.

Decreased MoCA scores were correlated with increased age, high urea, low eGFR, low high UACR, vitamin D, low Hb, low serum albumin, high CRP, and high GDS.

A multivariate analysis including all factors presenting a significant correlation in the bivariate analyses was performed.

In the multivariate analysis, age [B 95%CI −0.36 (−0.42; −0.30), *p* < 0.001], serum albumin [B 95%CI 1.86 (0.86; 2.85), *p* < 0.001], and vitamin D levels [B 95%CI 0.05 (0.02; 0.07), *p* = 0.002] remain significant, influencing the MOCA score. When age was not taken into the linear regression multivariate model as an independent variable, UACR [B 95%CI −0.003 (−0.005; −0.001), *p* = 0.014] was significant in predicting the MOCA score.

Increased GDS score was correlated with increased age, high urea, lower eGFR, low vitamin D, low Hb, low serum albumin, higher CRP, and lower MoCA score (Table 2).

In multivariate analysis, age [B 95%CI 0.05 (0.01; 0.09), *p* = 0.014] and vitamin D [B 95%CI −0.02 (−0.04; 0), *p* = 0.046] remained significant, influencing the GDS score. When age was not taken into the linear regression multivariate model as an independent variable, serum albumin [B 95%CI −0.82 (−1.52; −0.12), *p* = 0.022] and eGFR (transformed in ranks) [B 95%CI −0.002 (−0.003; 0), *p* = 0.014] (transformed as rank) were significant in predicting the GDS score.

## 4. Discussion

### 4.1. Prevalence of Mental and Kidney Involvement

The world is rapidly moving towards a hyperaged society, and the number of patients with cognitive impairment is increasing rapidly, along with the number of those with CKD [16]. Our cross-sectional analysis of geriatric inpatients identified an important burden of cognitive impairment and depression in the context of kidney dysfunction. We report a prevalence of 59.38% for CKD, 74.3% for cognitive impairment, and 62.4% for depression.

A previous study had shown that CKD is more common in elderly hospitalized patients, and among CKD individuals, hospitalization and mortality were increased compared with non-elderly patients [17]. Previous studies reported that cognitive dysfunction and dementia are strongly associated with advanced age [18] and are common in patients with CKD [19]. The exact mechanisms underlying cognitive dysfunction in CKD are not fully understood, but factors such as vascular damage, inflammation, oxidative stress, and accumulation of uremic toxins may have a role [18,19]. Studies suggest depression is common among older adults, with CKD affecting up to 20–30% depending on the stage and whether the patient is on dialysis [20]. Previous studies identified significant associations involving non-dialysis CKD patients and their cognition, depression, and mental health outcomes [5,13]. We encountered higher prevalence explainable by the fact that we included hospitalized geriatric patients, and most of the studies report data from outpatient participants.

### 4.2. Cognitive Impairment and Kidney Dysfunction

An important finding of our study was the correlation between decreased eGFR and low MoCA. Accumulation of waste products due to impaired kidney filtration may have neurotoxic effects. Therefore, the brain–kidney axis concept was proposed to emphasize the roles of kidney functioning in modulating neurodegeneration. 

The study “Good Aging in Skåne” from Sweden explored the relationship between kidney function and cognitive function in the general older population. The results from the cross-sectional analysis indicated that low eGFR is associated with impaired function in various cognitive domains, but in their longitudinal study, eGFR was associated only with decline in processing speed, but not with incident dementia or cognitive impairment [21,22].

A systematic review and meta-analysis of a large adult population identified an association between the increased risk of dementia or cognitive decline and acute kidney injury, CKD, higher serum creatinine, higher UACR, and lower eGFR. Yet, due to heterogeneity, the authors concluded that this evidence warrants further investigation [23].

Some studies have suggested that cognitive disturbances can be detected even in mild or moderate CKD [5,6,24]. Our results indicate a relationship between CKD and cognitive impairment in elderly patients even in the case of minimal reduction in eGFR. Decreased eGFR was significantly associated with impairments in global cognition, but also in specific areas like memory, language, and executive function [25].

A recent case–control study evaluated the cognitive function in patients with CKD stages 3 and 4 using the MoCA test. The study reported cognitive dysfunction in 12.9% of the control group and 37.1% of the case group, which was statistically significant [26]. As in this study, our research identified more cases of cognitive impairment using MoCA, possibly because this tool is very sensitive and able to detect mild cognitive decline. When compared with other cognitive tests such as the Mini-Mental State Examination (MMSE), the MoCA has a higher sensitivity for mild cognitive impairment but less value for evaluation of moderate to severe dementia [6]. Studies have shown that MoCA scores tend to be lower in CKD patients, even before end-stage renal disease, and that lower eGFR is correlated with worse cognitive scores, especially in domains of attention and executive function [5].

In this context of potential neurocognitive deficit, some authors point out that, in advanced-stage CKD, the decision to undergo renal replacement therapy should not be made by the patient alone, as it may not be the correct approach, and a cognitive assessment is needed [25,26]. Screening older patients with chronic kidney disease (CKD) for cognitive impairment prior to initiating dialysis or transplant evaluation is particularly important, as it can help personalize treatment strategies, inform decisions between dialysis and conservative management, and support caregiver involvement and advance care planning. Moreover, managing renal function may represent a valuable avenue for predicting and potentially preventing dementia.

### 4.3. Cognitive Impairment and Albuminuria

We also identified a significant correlation between increased albuminuria and cognitive impairment. Albuminuria is probably a better indicator of CKD than reduced eGFR, as it also reflects general dysfunction of the vascular and glomerular endothelium [27]. But albuminuria was also associated with cardiovascular disease, reflecting low-grade systemic inflammation and endothelial dysfunction. Albuminuria indicates microvascular damage in the kidneys. A nationwide, population-based cohort study, including adults in Denmark with incident CKD stage G3, demonstrated that more severe albuminuria is a risk factor for 3-year risks of rapid progression [28]. Nevertheless, albuminuria as a marker of microvascular disease often mirrors a similar pathology in the brain, leading to cognitive issues. Moreover, in elderly patients, albuminuria was associated with cognitive dysfunction independently of small vessel disease [29]. Also noteworthy is the Hisayama Study, which revealed significant associations between albuminuria, kidney dysfunction, and incident dementia. Albuminuria demonstrated associations with both Alzheimer’s disease and vascular dementia. In contrast, kidney dysfunction, as indicated by an eGFR of <60 mL/min/1.73 m^2^, was specifically associated only with vascular dementia but not linked to the development of Alzheimer’s disease [30].

Changes in albuminuria are usually used as surrogate endpoints for progression of kidney disease and the efficacy of different CKD treatments [31]. As cognitive decline proved to be common in patients with CKD and correlated with urinary albumin loss, we may hypothesize that albuminuria may serve as a biomarker for cognition. As a result, regular screening for albuminuria in elderly patients with cognitive impairment may be a good tool, and the use of diet and drugs for reducing albuminuria could offer neuroprotection. Consequently, albuminuria in geriatric patients is more than just a marker of kidney dysfunction; it is a red flag for potential cognitive decline. Proactive screening and multidisciplinary care of the kidney–brain axis can improve outcomes in this vulnerable population [32].

### 4.4. Depression and Kidney Dysfunction

Depression is common in CKD and is associated with a significant risk of adverse outcomes, including increased mortality and hospitalization rates, as well as poorer treatment compliance and quality of life [33]. In our study, worsening kidney insufficiency was correlated with higher depression severity.

Clinical evaluation of depression in elderly patients with CKD can be challenging. However, there are trustworthy evaluation tools such as the GDS score, and once the diagnosis is made, a range of treatment modalities can be considered. The use of GDS in CKD geriatric patients is especially relevant due to the high rate of undiagnosed and untreated depression in this population due to lack of awareness and screening. Studies have validated the GDS in CKD and dialysis patients, finding it sensitive and specific for detecting depression [33].

Depression adversely impacts health outcomes, elevates the risks of non-adherence to treatments and suicide, contributes to cognitive impairment, and predicts mortality among patients with CKD. Depression and anxiety also substantially reduce patient wellbeing, especially among the older CKD population. Given the importance of this issue, there is now an urgent need for well-conducted randomized trials of interventions for depression in CKD to provide information on the safety and efficacy of treatments. Depression and CKD in the elderly combine in an adverse combination impacting functionality, quality of life, and survival; therefore, efforts have to be made considering our continuously aging society [13,20].

Certain therapeutic solutions, particularly those with minimal pharmacological impact, such as physical activity, can be highly beneficial for patients with CKD. Since depression often leads to physical inactivity, engaging in exercise not only improves biological outcomes but also enhances psychiatric well-being. Recent studies have shown that exercise interventions significantly reduce depressive symptoms in CKD patients, with a systematic review and meta-analysis reporting moderate reductions in depression [34]. Additionally, exercise training, including intradialytic and aerobic exercises, has been shown to reduce depression levels in hemodialysis patients [35].

Several studies identified associations between albuminuria and depression. In the Maastricht Study, albuminuria was associated with a higher prevalence of depression in a population-based cohort [36]. In healthy young people, suicidal ideation was associated with increased albuminuria, as shown by a national cross-sectional study performed in Korea [37]. In our study, depression scores were not correlated with the levels of albuminuria. An interesting study from Norway reported positive and significant associations between moderately increased albuminuria and symptoms of depression in unadjusted analyses, but significance disappeared after adjustments [38]. Also, in their longitudinal 10-year follow-up study, this research group found no statistical evidence for an association between baseline depressive symptoms and subsequent albuminuria, nor between baseline albuminuria and subsequent depressive symptoms [39]. Although our patients with CKD defined by low eGFR or albuminuria had depression more often, considering that we have not found any correlation between albuminuria and depression, we hypothesize that albuminuria probably reflects another comorbidity or inflammatory condition than depression symptomatology.

When age was not taken into the linear regression multivariate model as an independent variable, albuminuria was significant in prediction of cognitive impairment, and low eGFR was significant in prediction of depression. These results highlight the role of kidney involvement diagnostic markers as risk factors for mental health.

### 4.5. Other Factors with Influence on Mental Health in Elderly Patients

Other findings of our study were the correlations between declining cognitive status and depression, low serum albumin, and high serum CRP, confirming the results of previous studies. Chronic low-grade inflammation in elderly patients may contribute to neurodegeneration, and there is an ongoing debate on the contribution of inflammation to neuro-psychiatric disorders [40].

Anemia and hypovitaminosis D were also correlated with cognitive decline and depressive symptoms. These are common consequences of CKD, and each one can have an impact on mental health. Anemia-induced hypoxia increases the production of reactive oxygen species via mitochondrial dysfunction, which directly harm endothelial cells, increase vascular permeability, and compromise the integrity of the blood–brain barrier with clinical consequences for different brain functions, increasing the cognitive vulnerability [41,42]. Hypovitaminosis D has been linked to serious brain functionality deficits. Calcitriol enhances learning, memory, and exploratory behavior in animal models, indicating improved cognitive abilities. These effects are often linked to increased expression of proteins contributing to synapse formation and stabilization [43,44]. Nutrient deficiencies are common in people with CKD, including low levels of essential vitamins such as vitamin D and B vitamins, all of which are crucial for maintaining brain health and cognitive function [45]. A recent cohort study demonstrated an independent association between vitamin D deficiency and increased depression risk in patients aged ≥50 years with CKD, particularly in males [46].

*This study had a few limitations.* First, data on CKD etiology, which can influence kidney function and urinary albumin loss, were lacking. Second, specific risk factors for mental health deterioration were not analyzed. Third, the study included a small number of participants with severe kidney dysfunction, and therefore it was not possible to study the relationship between severe kidney disease and the development of cognitive impairment and depression. Finally, this study was cross-sectional and observational in design, so causal relationships and long-term clinical outcomes could not be confirmed.

Although it has some limitations, our study analyzed a large cohort of elderly people and identified an important number of patients with CKD and brain problems. In addition, our study analyzed two modalities to assess mental health and highlighted that cognitive impairment and depression are correlated with decreased kidney function, and cognitive impairment is correlated with albuminuria. The relationship between cognitive decline and albuminuria has been relatively less studied in older patients. Finally, although the study was cross-sectional, our findings gained added value due to the substantial sample and the high significance of the results.

## 5. Conclusions

In conclusion, cognitive decline and depression were highly prevalent among older hospitalized patients. Cognitive dysfunction was associated with both elevated albuminuria and reduced eGFR, while depressive symptoms were more pronounced in individuals with lower eGFR but showed no association with albuminuria. This study provides new insights into the complex interplay between cognitive impairment, depression, albuminuria, and kidney dysfunction in the elderly population. The convergence of aging, kidney dysfunction, and mental health disorders creates a vulnerable population requiring comprehensive assessment and management strategies. Personalized cognitive and depression evaluation plans should be integrated into the care of old CKD patients, even to mild increase of albuminuria levels with a definitive purpose to prevent mental health decline effectively. Therefore, public health strategies that focus on the early identification and management of mental and cognitive impairments in CKD patients are strongly recommended.

## Figures and Tables

**Table 1:** The characteristics of the patients from the study cohort and the comparison between groups.

Parameter	Parameter Categories	All (n = 719)	eGFR ≥ 60 mL/min/m^2^ and UACR < 30 mg/g (n = 292)	eGFR < 60 mL/min/m^2^ or UACR ≥ 30 mg/g (n = 427)	*p*
Age		80 (74; 85)	77 (71; 82)	82 (76; 86)	**<0.001**
Male, no. (%)		214 (29.8)	99 (33.9)	115 (26.9)	**0.045**
Diabetes, no. (%)		246 (34.3)	88 (30.2)	158 (37.1)	0.058
Urea (mg/dL)		47 (39; 62)	41 (34; 50)	54 (43; 75)	**<0.001**
Creatinine (mg/dL)		0.91 (0.74; 1.17)	0.77 (0.69; 0.88)	1.09 (0.88; 1.37)	**<0.001**
eGFR (mL/min/m^2^)		69.16 (47.46; 86.32)	84.65 (73.09; 93.43)	50.91 (37.82; 72.42)	**<0.001**
Uric acid (mg/dL)		6 (4.9; 7.2)	5.4 (4.3; 6.5)	6.5 (5.2; 7.9)	**<0.001**
UACR (mg/g)		19.35 (10.38; 52.76)	11.68 (8.49; 16.96)	41.11 (15.94; 106.79)	**<0.001**
Vitamin D (ng/mL)		22.9 (14.31; 32.71)	24.7 (15.97; 33.61)	21.12 (13.08; 32.38)	**0.005**
Hb (g/dL)		12.8 (11.5; 13.9)	13.2 (12; 14.2)	12.5 (11.1; 13.5)	**<0.001**
Vitamin B12 (pg/mL)		337.15 (246.54; 476.81)	321.13 (232.79; 442.74)	349.76 (257.6; 512.18)	**0.012**
Albumin (g/dL)		4.2 (4; 4.5)	4.3 (4; 4.6)	4.2 (3.9; 4.5)	**<0.001**
CRP (mg/dL)		0.48 (0.18; 1.6)	0.34 (0.15; 0.84)	0.58 (0.23; 2.15)	**<0.001**
MoCA Score		21 (16; 26)	22 (17; 27)	21 (15; 25)	**0.001**
MoCA < 26, no. (%)		532 (74)	197 (67.5)	335 (78.5)	**0.001**
GDS Score		6 (3; 10)	5 (3; 9)	7 (4; 10)	**0.007**
GDS > 4, no. (%)		445 (61.9)	162 (55.5)	283 (66.3)	**0.003**

Data are expressed as median (25th–75th percentile) or numerical value (percentages). eGFR, estimated glomerular filtration rate; UACR, urinary albumin over creatinine ratio; CRP, C-reactive protein; Hb, hemoglobin; MoCA, Montreal Cognitive Assessment; GDS, geriatric depression scale. Bold highlights the statistically significant results.

**Table 2:** Correlations between MoCA score, GDS score, and study variables—the bivariate analysis.

	MoCA Score (n = 719)		GDS Score (n = 719)	
	Spearman Correlation Coefficient	*p*	Spearman Correlation Coefficient	*p*
Age	−0.479	**<0.001**	0.155	**<0.001**
Urea (mg/dL)	−0.135	**<0.001**	0.116	**0.002**
Creatinine (mg/dL)	−0.057	0.125	0.057	0.127
Uric acid (mg/dL)	−0.049	0.198	0.033	0.379
eGFR (mL/min/m^2^)	0.123	**0.001**	−0.131	**<0.001**
UACR (mg/g)	−0.155	**<0.001**	0.038	0.314
Vitamin D (ng/mL)	0.247	**<0.001**	−0.107	**0.004**
Hb (g/dL)	0.143	**<0.001**	−0.098	**0.009**
Vitamin B12 (pg/mL)	−0.034	0.359	0.053	0.155
Albumin (g/dL)	0.283	**<0.001**	−0.143	**<0.001**
CRP (mg/dL)	−0.126	**0.012**	0.179	**<0.001**
MoCA Score	-	**-**	−0.369	**<0.001**
GDS Score	−0.369	**<0.001**	-	-

eGFR, estimated glomerular filtration rate; UACR, urinary albumin over creatinine ratio; Hb, hemoglobin; CRP, C-reactive protein; MoCA, Montreal Cognitive Assessment; GDS, geriatric depression scale. Bold highlights the statistically significant results.

## Data Availability

The research data supporting this study’s findings are not publicly available. Further inquiries can be directed at the corresponding author. The dataset used during the current study is available from the corresponding authors upon reasonable request.
